# Supplementary figures and images for: Deep learning approaches to landmark detection in tsetse wing images
Source: PLoS Comput Biol. 2023 Jun 26;19(6):e1011194. doi: 10.1371/journal.pcbi.1011194 (PMC10328335; doi:10.1371/journal.pcbi.1011194)

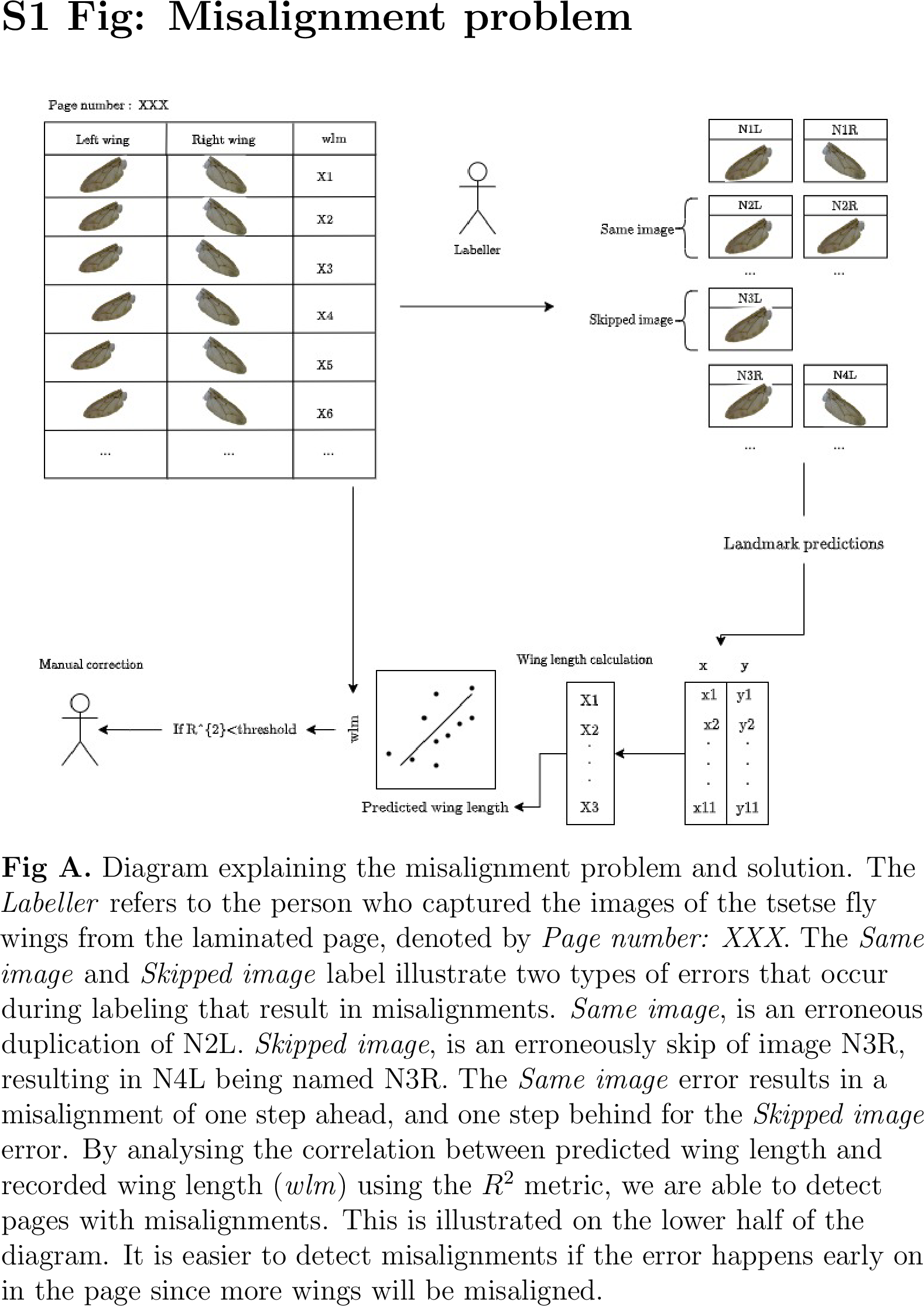

Supplement: S1 Fig — Fig A. Diagram explaining the misalignment problem and solution. The Labeller refers to the person who captured the images of the tsetse fly wings from the laminated page, denoted by Page number: XXX. The Same image and Skipped image label illustrate two types of errors that occur during labeling that result in misalignments. Same image, is an erroneous duplication of N2L. Skipped image, is an erroneously skip of image N3R, resulting in N4L being named N3R. The Same image error results in a misalignment of one step ahead, and one step behind for the Skipped image error. By analysing the correlation between predicted wing length and recorded wing length (wlm) using the R2 metric, we are able to detect pages with misalignments. This is illustrated on the lower half of the diagram. It is easier to detect misalignments if the error happens early on in the page since more wings will be misaligned. (TIF) [file pcbi.1011194.s001.tif]

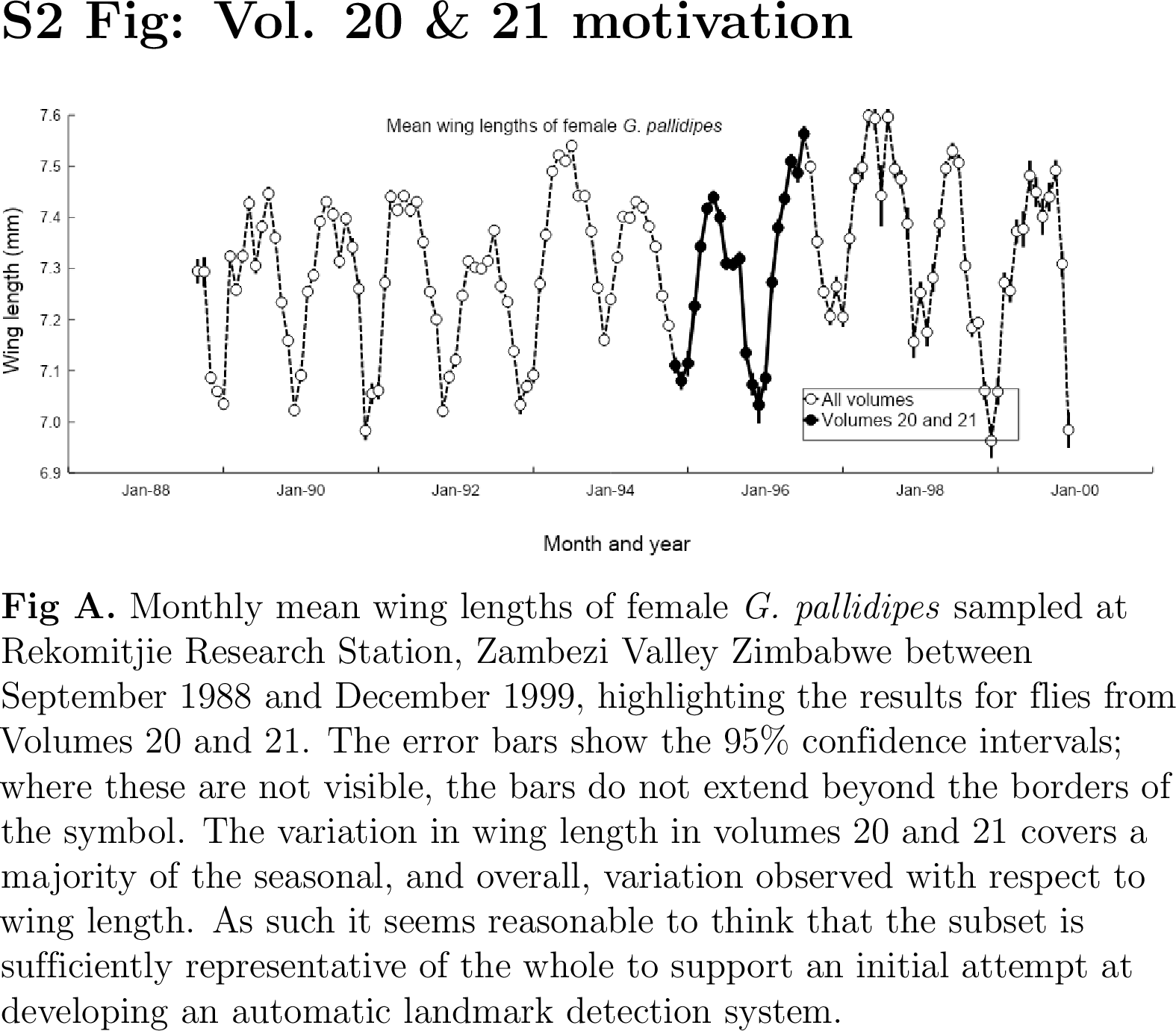

Supplement: S2 Fig — Fig A. Monthly mean wing lengths of female G. pallidipes sampled at Rekomitjie Research Station, Zambezi Valley Zimbabwe between September 1988 and December 1999, highlighting the results for flies from Volumes 20 and 21. The error bars show the 95% confidence intervals; where these are not visible, the bars do not extend beyond the borders of the symbol. The variation in wing length in volumes 20 and 21 covers a majority of the seasonal, and overall, variation observed with respect to wing length. As such it seems reasonable to think that the subset is sufficiently representative of the whole to support an initial attempt at developing an automatic landmark detection system. (TIF) [file pcbi.1011194.s002.tif]

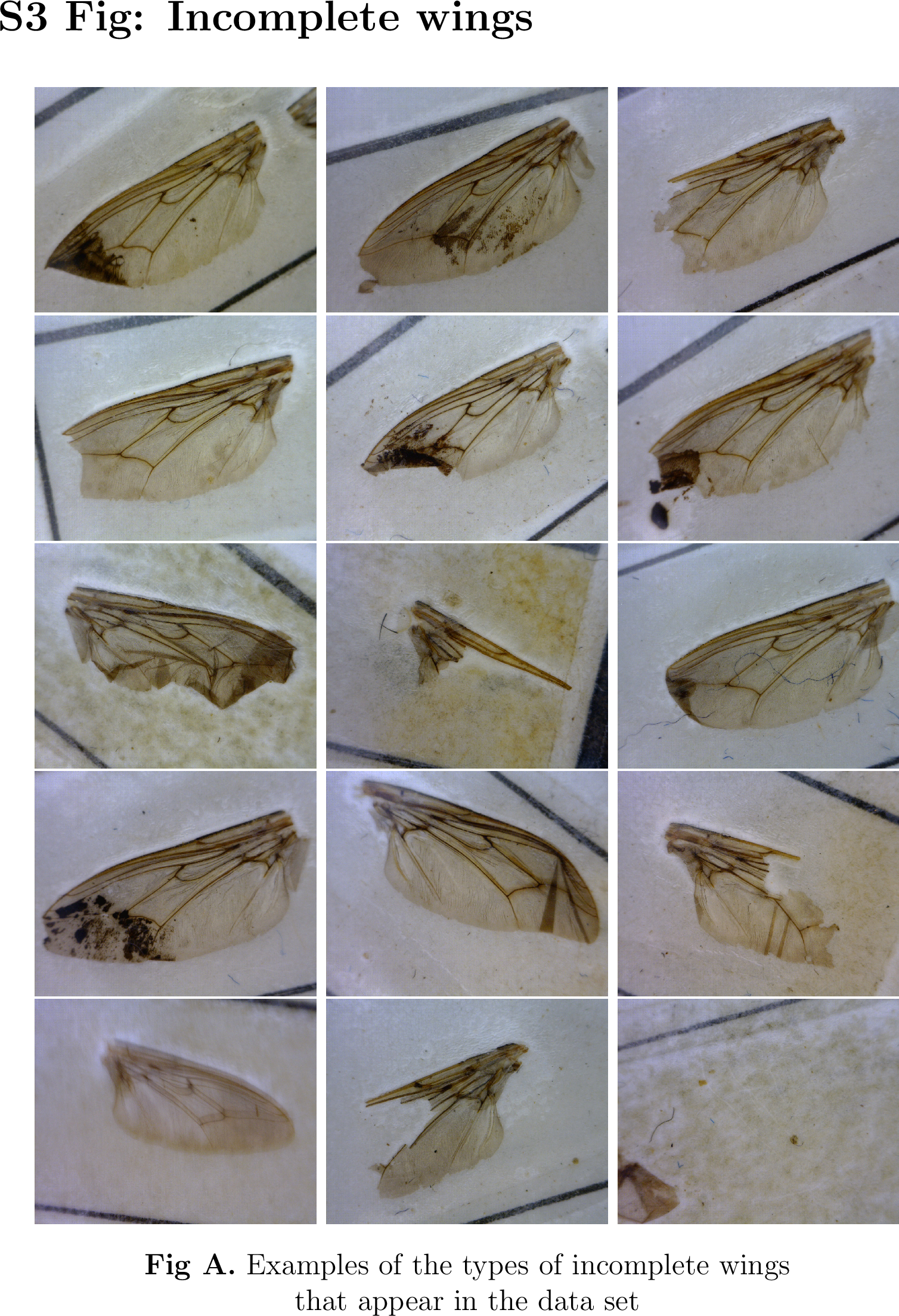

Supplement: S3 Fig — Fig A. Examples of the types of incomplete wings that appear in the data set. (TIF) [file pcbi.1011194.s003.tif]

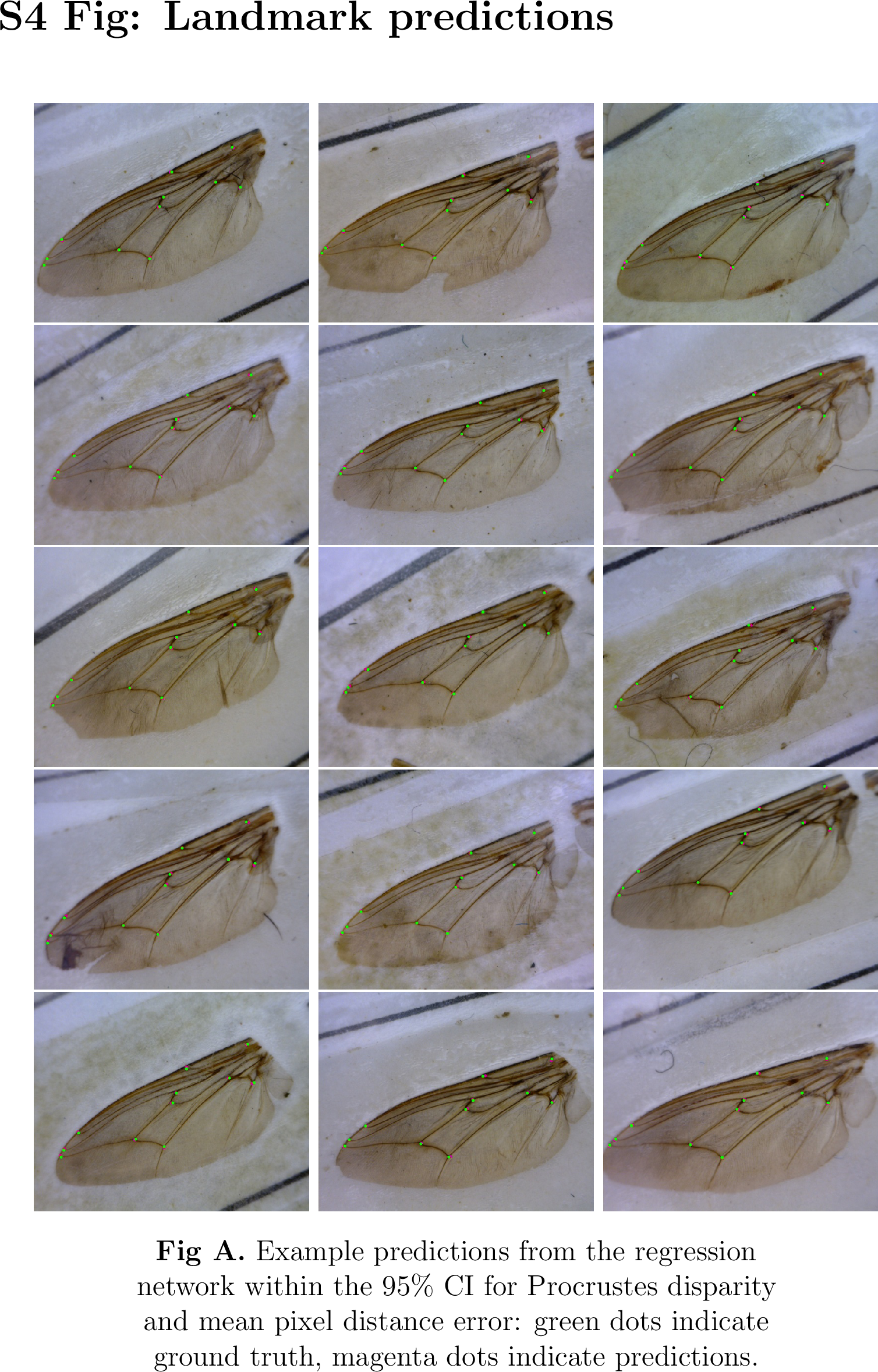

Supplement: S4 Fig — Fig A. Example predictions from the regression network within the 95% CI for Procrustes disparity and mean pixel distance error: green dots indicate ground truth, magenta dots indicate predictions. (TIF) [file pcbi.1011194.s004.tif]
