# Supplementary material for: Deep learning approaches to landmark detection in tsetse wing images
Source: PLoS Comput Biol. 2023 Jun 26;19(6):e1011194. doi: 10.1371/journal.pcbi.1011194 (PMC10328335; doi:10.1371/journal.pcbi.1011194)
Supplement: S1 Text — Table A. The variable names given in this table were recorded during the dissection of each respective fly. Note that these variables are only some of the variables available for each fly which are given as part of the morphometric data set published with this research. (DOCX) [file pcbi.1011194.s005.docx]

S1 Text: Biological recordings

| **Data captured during lab dissection** | |
| --- | --- |
| **Variable name** | **Description** |
| vpn | Volume, page and number |
| cd | Day of the month that fly was recorded |
| mc | Month of the year that fly was recorded |
| cy | Year that fly was recorded |
| md | Method used to capture fly |
| g | Genus of the fly [Gp = G. pallidipes or Gmm = G. m. morsitans] |
| s | Sex [1= Male 2 = Female] |
| c | Ovarian category, which measures age [0 to 7] |
| wlm | wing length |
| f | Wing fray category, also measures age [1 – 6] |
| lmkl | Number of landmarks missing on LEFT wing [1 – 11] |
| lmkr | Number of landmarks missing on RIGHT wing [1 – 11] |
| hc | Index. 1 if hatchet cell used; 0 if distance landmarks 1 to 6 used |

**Table A.** The variable names given in this table were recorded during the dissection of each respective fly. Note that these variables are only some of the variables available for each fly which are given as part of the morphometric data set published with this research.
