# Supplementary material for: Deep learning approaches to landmark detection in tsetse wing images
Source: PLoS Comput Biol. 2023 Jun 26;19(6):e1011194. doi: 10.1371/journal.pcbi.1011194 (PMC10328335; doi:10.1371/journal.pcbi.1011194)
Supplement: S2 Text — Table A. VGG16, parameters: 1474822. Table B. ResNet18, parameters: 11177025. Table C. Inception V3, parameters: 24346082. Fig A. Loss per epoch, illustrating training stability and convergence speed. (DOCX) [file pcbi.1011194.s006.docx]

S2 Text: Classification scores

Tables A, B and C provide the score and confidence intervals for each metric score. Tables B and C show that ResNet18 and Inception have a trade-off between specificity and sensitivity, with ResNet18 having a better specificity and Inception a better sensitivity. The best performing was VGG16 with batch normalisation, attaining 100% on all metrics.

| 95% confidence interval | specificity | sensitivity | precision | f1 score | accuracy |
| --- | --- | --- | --- | --- | --- |
| lower bound | 1.000 | 1.000 | 1.000 | 1.000 | 1.000 |
| value | 1.000 | 1.000 | 1.000 | 1.000 | 1.000 |
| upper bound | 1.000 | 1.000 | 1.000 | 1.000 | 1.000 |

**Table A.** VGG16, parameters: 14748225

| 95% confidence interval | specificity | sensitivity | precision | f1 score | accuracy |
| --- | --- | --- | --- | --- | --- |
| lower bound | 1.000 | 0.972 | 1.000 | 0.986 | 0.985 |
| value | 1.000 | 0.993 | 1.000 | 0.991 | 0.990 |
| upper bound | 1.000 | 1.00 | 1.000 | 1.000 | 1.000 |

**Table B.** ResNet18, parameters: 11177025

| 95% confidence interval | specificity | sensitivity | precision | f1 score | accuracy |
| --- | --- | --- | --- | --- | --- |
| lower bound | 0.957 | 1.000 | 0.961 | 0.980 | 0.980 |
| value | 0.985 | 1.000 | 0.980 | 0.990 | 0.990 |
| upper bound | 1.000 | 1.000 | 1.000 | 1.000 | 1.000 |

**Table C.** Inception V3, parameters: 24346082

Training loss per epoch

Inception

ResNet18 VGG16

0*.*4

0*.*3

Binary Cross Entropy loss

0*.*2

0*.*1

0*.*0

0 2 4 6 8 10 12

Epoch

**Fig A.** Loss per epoch, illustrating training stability and convergence speed
