# Supplementary material for: Deep learning approaches to landmark detection in tsetse wing images
Source: PLoS Comput Biol. 2023 Jun 26;19(6):e1011194. doi: 10.1371/journal.pcbi.1011194 (PMC10328335; doi:10.1371/journal.pcbi.1011194)
Supplement: S3 Text — Table A. A table of sample statistics for pages containing misaligned data. Table B. A table detailing the amount of misaligned pages found and whether they were corrected or removed from the data set. (DOCX) [file pcbi.1011194.s007.docx]

# S3 Text: Data information

## Sample statistics for misaligned pages

| Sample size | 100 |
| --- | --- |
| Population size | 770 |
| Confidence interval | 95% |
| Pages found (%) | 2 |
| Margin of error (%) | 2.56 |

**Table A.** A table of sample statistics for pages containing misaligned data

| Number of pages containing misaligned data | 15 |
| --- | --- |
| Number of pages corrected | 12 |
| Number of pages removed | 3 |

**Table B.** A table detailing the amount of misaligned pages

found and whether they were corrected or removed from the data set.

## Alterations to volume 20 and 21

Pages with *R*^2^*<* 0.1 :

- 3 pages removed - 36, 48, 321
- 12 pages corrected - 11, 34, 35, 42, 75, 159, 187, 201, 226, 232, 357,

358.

## Directions for morphometric studies using these landmarks

The principal motivation for studies of tsetse wing morphometrics was to provide phenotypic measures that could be used to gauge whether given populations of tsetse were sufficiently different that they could be considered isolated from each other, such that a single population could be eradicated without fear of reinvasion from neighbouring populations. It has been suggested that wing morphology is a good marker for typifying tsetse populations [[1].](#_bookmark0) The utility of morphometric analysis in support of such analyses will depend, however, on the extent to which the variation and covariation in wing shape and size depends on environmental factors. If wing morphometry changes substantially, with climate and season, within a single isolated population, such environmentally induced changes might be sufficient to mask the differences between geographically distant populations of tsetse.

Such issues have not been studied for tsetse, mainly because sample sizes have been too small and collected over too short a time period. The collection of wings which provided the subset used in the present analysis does allow for the possibility of studying morphometric changes over a long period of time, at all seasons, in flies that were all collected within 2 km of a single site, Rekomitjie Research Station in the Zambezi Valley of Zimbabwe. As such the collection provides a valuable resource for studying the natural variation in wing morphometrics of a single tsetse population.

The biological data (obtained via lab dissection) used in this study has already been used to show that wing lengths change significantly, and consistently with season in both G. pallidipes and G. m. morsitans [[2].](#_bookmark1) Similar analyses now need to be carried out on the extent of changes in the morphometry of the same wings. As with the study on the effect of season on changes in wing length, it is important in carrying out analyses of morphometry to allow both for the time of capture of the flies in question and also for the age of the fly in question. This is because tsetse are relatively long-lived compared with most insects. Consequently, a fly captured at a time of year when temperatures are favourable for survival, might have developed as a pupa, and emerged as an adult when conditions were much less favourable. For female tsetse in the data set, there are data on the ovarian age of the flies at capture, so that we will be able to allow for such variation. For males we clearly cannot have such measures, but we do at least have data on wing fray that can be used to give approximate values of the age. In conclusion, our data set provides a rich resource for carrying out detailed analyses of the factors affecting wing shape in tsetse.

# References

1. Patterson JS, Schofield CJ. Preliminary study of wing morphometry in relation to tsetse population genetics. South African Journal of Science. 2005;101:132-134.
2. Hargrove JW, English S, Torr SJ, Lord J, Haines LR, Van Schalkwyk C, et al. Wing length and host location in tsetse (Glossina spp.): implications for control using stationary baits. Parasites & Vectors. 2019 Dec;12(1):1-3.
