# Supplementary material for: Deep learning approaches to landmark detection in tsetse wing images
Source: PLoS Comput Biol. 2023 Jun 26;19(6):e1011194. doi: 10.1371/journal.pcbi.1011194 (PMC10328335; doi:10.1371/journal.pcbi.1011194)
Supplement: Attachment — Submitted filename: PCOMPBIOL-D-22-00611_reviewer_01.pdf [file pcbi.1011194.s008.pdf]

Geldenhuys et al. present a two-tier method using deep learning architectures to classify images and make accurate landmark predictions of tsetse fly wings. While the first tier removed images with missing data/landmark points, the second tier provides landmarks of the remaining images. The manuscript is well written and includes outstanding description of data, misalignment / potential error, outliers and erroneous inliers.
While the overall manuscript writing and style is noticeable good, my main critique is that the actual rationale of the paper is buried in the supplementary "*Directions for morphometric studies using these landmarks*". Thus, only at the very end, the actual meaningfulness and future applications become impactful.

Main comments:

i. The manuscript would greatly benefit from extracting section of the supplementary "*Directions for morphometric studies using these landmarks*" and their inclusion throughout appropriate sections in abstract, introduction, and discussion. This is ultimately the true societal impact of this study. Additionally, the manuscript would benefit from highlighting that future impactful work will rely on relating the here analysed data with biological findings.

ii. The authors touch on applicability to other species and how their study relates to other findings. From a biological perspective, it would make sense to merge the 2nd and 2nd-to-last paragraph of the discussion and potentially expand on this. This would make the study more meaningful, especially for the Drosophila field.

iii. Are the data actually from: "Hargrove, J., English, S., Torr, S.J. et al. Wing length and host location in tsetse (Glossina spp.): implications for control using stationary baits. Parasites Vectors 12, 24 (2019). https://doi.org/10.1186/s13071-018-3274-x"? This isn't clear and needs to be clarified throughout.

iv. The authors talk a lot about image quality in terms of anatomical features. But could they expand a bit more on image quality with respect to for example noise, signal-to-noise ratio etc. – Can the authors comment on whether data augmentation for these image properties could further increase their analysis outcomes?

v. Reference [40] is currently "*Data Dryad Tsetse landmarks for volumes 20 and 21 TODO*" – please rectify.

vi. Reference [33] refers to your code. Please acquire a code doi (https://docs.github.com/en/repositories/archiving-a-github-repository/referencing-and-citing-content) and reference accordingly.

vii. Please include appropriate repository and code description on the GitHub page (https://github.com/DylanGeldenhuys/Landmark-detection-for-tsetse-fly-wings). There also seem to be some errors in your Jupyter Notebooks – please rectify.

Minor:

i. Interesting data on incomplete wings are currently in the Material & Methods section, I would move them to the Results.

ii. Fig 2 is too small. Colours would help to identify steps and what the intention of the bottom half is.
iii. To facilitate wider uptake, I would maybe mention in abstract that the code is in Python (i.e. open-source and non-commercial) and already available.
iv. For Suppl Fig 1, please change from red-green points to magenta-green to be colour-blind friendly (apply to any other data where applicable).
v. Please add a scale bar with microns to Fig 1.
vi. Line 51 – a reference is not correctly compiled, resulting in [?,..]
vii. Line 154 – currently “i” in “incomplete” where a capital should be.
