# Supplementary material for: Deep learning approaches to landmark detection in tsetse wing images
Source: PLoS Comput Biol. 2023 Jun 26;19(6):e1011194. doi: 10.1371/journal.pcbi.1011194 (PMC10328335; doi:10.1371/journal.pcbi.1011194)
Supplement: Attachment — Submitted filename: response_to_reviewers.pdf [file pcbi.1011194.s009.pdf]

Addressing review remarks

Reviewer 1

Geldenhuys et al. present a two-tier method using deep learning architectures to classify images and make accurate landmark predictions of tsetse fly wings. While the first tier removed images with missing data/landmark points, the second tier provides landmarks of the remaining images. The manuscript is well written and includes outstanding description of data, misalignment / potential error, outliers and erroneous inliers.
While the overall manuscript writing and style is noticeable good, my main critique is that the actual rationale of the paper is buried in the supplementary “Directions for morphometric studies using these landmarks”. Thus, only at the very end, the actual meaningfulness and future applications become impactful.

Main comments:

i. The manuscript would greatly benefit from extracting section of the supplementary “Directions for morphometric studies using these landmarks” and their inclusion throughout appropriate sections in abstract, introduction, and discussion. This is ultimately the true societal impact of this study. Additionally, the manuscript would benefit from highlighting that future impactful work will rely on relating the here analysed data with biological findings.

We thank Reviewer 1 for this insightful and helpful comment. It is clear to us that, in writing this paper, we have been too closely focused on the technical problems involved – to the detriment of contextualising the paper relative to the biology of tsetse, and to the control of the flies and of the diseases they transmit.

Accordingly, as suggested by Reviewer 1, we have moved out of the Supplementary Material section headed “Directions for morphometric studies using these landmarks” and used the material in entirely reworking the Abstract, Authors summary and Introduction.

Moreover, we have added several paragraphs at the end Discussion, suggesting how the current morphometric analyses, combined with other biological findings, will impact our understanding of tsetse biology and our ability to control the flies and the diseases they spread.

ii. The authors touch on applicability to other species and how their study relates to other findings. From a biological perspective, it would make sense to merge the 2nd and 2nd-to-last paragraph of the discussion and potentially expand on this. This would make the study more meaningful, especially for the Drosophila field.

We thank the reviewer for this comment; another reviewer also commented on this section, explaining that our claim, that our method is stronger, is weak since we did not test it on their data. We removed this claim and explained how our method could transfer to the drosophila data sets mentioned as follows on lines 519-528:

- “Loosely comparing our results with former mentioned studies, Vandaele et al. reported a similar mean pixel distance error of approximately 6 pixels for images of a smaller size 1440X900. Porto et al. reported a mean pixel distance error of 0.57%, normalising the mean pixel distance by the largest wing length. We obtained a smaller normalised pixel distance error of 0.47% for the deployed regression model using the same metric. The Drosophila data sets are arguably less complex than our data, which often contains image artefacts and varying wing quality that may affect the complexity of the task. Therefore we argue that successfully applying transfer learning could yield better results on these Drosophila data sets.”

iii. Are the data actually from: “Hargrove, J., English, S., Torr, S.J. et al. Wing length and host location in tsetse (Glossina spp.): implications for control using stationary baits. Parasites Vectors 12, 24 (2019). https://doi.org/10.1186/s13071-018-3274-x”? This isn’t clear and needs to be clarified throughout.

We appreciate the reviewer's comment. This data has been used in the study mentioned. We have adjusted the wording in the supplementary material to make this more apparent on.

- “The biological data (obtained via lab dissection) used in this study has already been used to show that wing lengths change significantly, and consistently with season in both G. pallidipes and G. m. morsitans [32].”

iv. The authors talk a lot about image quality in terms of anatomical features. But could

they expand a bit more on image quality with respect to for example noise, signal-to-noise ratio etc. – Can the authors comment on whether data augmentation for

these image properties further increase their analysis outcomes?

We thank the reviewer for this consideration. Apart from the anatomical noise, in this study, we introduced image noise by adding affine transformations, which we observed more frequently upon inspection. Adding other types of noise could increase the model's robustness, but is less likely to have significant improvements since we already focus on addressing the more noisy parts of the data to ensure the model maintains a high signal-to-noise ratio in these cases. For example, adding colour noise may increase robustness. However, we argue that most of the noise in the data is due to affine transformations, which we address by adding various data augmentations during training. We have elaborated on this in the manuscript, referring to the signal-to-noise ratio in the section "Training implementation details" on line 274-283.

- In addition, we used image augmentations during all first-tier training and experimented with and without augmentations for the second tier training to improve model robustness. The augmentations increased the noise and size of the training data, preventing over-fitting and ensuring that the training data contained images on the extreme of the spectrum for wings available in volumes 20 and 21, in terms of wing location, rotation, and size which comprised most of the noisy characteristics of the data. These data augmentations helped maintain a high signal-to-noise ratio in the above mentioned noisy cases. We randomly sampled from a[-5%,5%] interval for scaling and shifting, and a [-22,22] interval for rotation to transform each image.

v. Reference [40] is currently “Data Dryad Tsetse landmarks for volumes 20 and 21

We have made the data publicly available and now referenced it in the article as:

Geldenhuys, Dylan (2022), Tsetse fly wing landmark data for morphometrics (Vol 20,21), Dryad, Dataset, https://doi.org/10.5061/dryad.qz612jmh1

vi. Reference [33] refers to your code. Please acquire a code doi (https://docs.github.com/en/repositories/archiving-a-github- repository/referencing-and-citing-content) and reference accordingly.

We thank the reviewer for bringing this to our attention, we have created a dio and referenced the repo as:

Geldenhuys D, Josias S. Landmark detection for tsetse fly wings [Computer software] 2022. DOI: 10.5281/zenodo.7199970

vii. Please include appropriate repository and code description on the GitHub page (https://github.com/DylanGeldenhuys/Landmark-detection-for-tsetse-fly-wings).
There also seem to be some errors in your Jupyter Notebooks – please rectify.

We thank the reviewer for bringing this to our attention. We have created a DIO for the repo and will ensure all notebooks don't have any errors, and will include a readme file describing the code before publishing.

Minor:
i. Interesting data on incomplete wings are currently in the Material & Methods section, I would move them to the Results.

We thank the reviewer for bringing this to our attention. We have moved the results of the sample statistics to the results section on line 322-324.

- The sample statistics showed that 13%±4.63% of wings are incomplete. The majority of incomplete wings were missing landmark 4 or 6, making up 10%±4.13% of the data, comprising on average 77% of all incomplete wings.

ii. Fig 2 is too small. Colours would help to identify steps and what the intention of the bottom half is.

We thank the reviewer for this suggestion. It has also been mentioned that this figure is too sparse to be a single image; we have made it more detailed to explain the overall process of this study clearly.

iii. To facilitate wider uptake, I would maybe mention in abstract that the code is in Python (i.e. open-source and non-commercial) and already available.

We thank the reviewer for this suggestion. We have included in the abstract that the code is written in python and open sourced.

iv. For Suppl Fig 1, please change from red-green points to magenta-green to be colour-blind friendly (apply to any other data where applicable).

We appreciate the reviewer for bringing this to our attention; we have altered the colours for this figure which is now part of S6.

v. Please add a scale bar with microns to Fig 1.

We thank the reviewer for this suggestion; we have left the image as is to allow readers to quickly gauge how large the pixel errors are, but have added the conversion to mm in the caption. We hope this is sufficient.

vi. Line 51 – a reference is not correctly compiled, resulting in [?,..]

We thank the reviewer for bringing this to our attention. We have corrected this reference.

vii. Line 154 – currently “i” in “incomplete” where a capital should be.

We thank the reviewer for pointing this out, we have corrected this.

Reviewer 2:

This work proposes a two-step deep learning workflow for anatomical landmark detection in tsetse wings. It is evaluated on a large, original, dataset acquired and cleaned by co-authors that they plan to make available. The two main workflow steps are: 1) a classifier to identify images where landmarks are missing, 2) a landmark detection model to localize 11 interesting points (either based on regression or segmentation, with/without data augmentation). The two steps reused existing works, but the combination of the two is original. The methodology is applied on a bigger dataset and there are plans to make it available for future research.

The study is interesting and it represents quite a lot of work that could be useful for the community. But I think it would be important to better clarify the contribution, either in terms of methodology, or in terms of dataset, or both. Here are my main comments:

We agree with Reviewer 2 that we need to clarify our contribution – as indeed we have noted above in responding to a comment from Reviewer 1. The following material has been added to the Discussion in addressing their comments (lines 539-571):

"Implications for studies on the biology and control of tsetse and trypanosomiasis, and morphometric studies on wings of all insects

If, as suggested in the literature [3-10], differences in wing shape between geographically separated populations of tsetse indicate the amount of genetic flow between such populations, it is crucial to ascertain the natural variability in wing shape at any given location. The study that provided wings for the current work affords that possibility – flies being captured in 136 consecutive months, at sampling sites all within a 2km radius of Rekomitjie Research Station in the Zambezi Valley of Zimbabwe. The females captured were subject to ovarian dissection to assess their age, pregnancy state, sperm content and mortality and abortion rates [15, 17, 24-26, 29, 33]. Large subsets were also dissected to identify their trypanosome infection status [14, 16, 18, 23, 34] and were subjected to nutritional analysis to assess fat, dry weight and residual blood-meal content [13, 19-22, 27, 28, 30]. Of particular interest are two papers assessing wing length and the extent of wing fray [31, 32]. We will therefore be able to examine the relationship between wing shape and a wide variety of biological attributes of the tsetse sampled.

After a tsetse wing expands and hardens, immediately post-adult-emergence, its length does not change. [21, 22, 31]. We assume that the shape will, similarly, be independent of the fly's age – but we will be able to check this using large samples of females where the age, and the date of birth, have been determined. Of importance will be an examination of the extent to which wing shape changes with the season and with climate variability between years across the 11 years of the study and, particularly, with the climatic conditions that a fly's mother experiences during pregnancy.

These detailed examinations of the way wing morphometrics data meshes with the underlying data set are beyond the scope of the present study, which simply aimed to establish the viability of a semi-automatic method for locating wing landmarks. The very good agreement we found between the wing lengths predicted in our study, and those measured manually using a binocular microscope in the underlying study. Fig 7 suggests that our method should be applied to all the wings we have available ourselves, and should allow other students of tsetse populations to carry out morphometric studies on much larger samples of wings than has been feasible until now. Of more general significance is the question of whether the method is applicable to the wings of other insects, a matter that we must allow other workers to decide."

For a methodological contribution, I think authors should extend their empirical evaluation to other methodologies and provide more precise empirical results. I would suggest to provide landmark prediction results without the first step (classifier) which are expected to be much lower, as a first baseline. Second, after the classification step, a second baseline could be a simple model that predicts the mean of landmark positions computed on the training set. For the segmentation approach, only a binary "disk centred at the (x,y) position of the landmark" (page 208) is evaluated while one could evaluate different heatmap generation strategies

(e.g. Gaussian or exponential functions based heatmaps such as in Yeh et al., Deep learning approach for automatic landmark detection and alignment analysis in whole-spine lateral radiographs. Scientific reports, 2021). A table with empirical results of these variants would help.

We thank the reviewer for the comments. We agree that our models perform very poorly when tasked to provide landmark predictions on incomplete wings. Although it would be an excellent exercise to predict landmarks for incomplete wings, we only intended to create predictions on complete wings because morphometric analysis requires a consistent amount of landmarks for most applications (e.g. Procrustes analysis), in addition the assumption of our models is that there are always 11 landmarks.

The suggestion for trying a model that predicts the mean of landmark positions from the training set is a neat way of comparing how much better the models perform compared to the most straightforward solution. We suggest that the models we present are baseline models, i.e. a good starting point for further development.

Concerning the heatmap variation, this is a great next step to try; however, we aimed to cover two different architectures generally used for different learning tasks. i.e. CNN for regression and FCN for classification. Including heatmap regression would introduce FCN with regression; this is worth trying and, based on recent papers, will most likely perform well, but we leave this for future work.

- It is also very important to clarify the empirical evaluation protocol. First, it is stated that "the study considers a data set containing 14354 pairs of images" (line 16), then authors mention they use a "one-fold cross-validation strategy" but this is a weird name for what seems to be a single independant train-validation-test split (because k-fold cross validation on 1 fold would mean dividing the full dataset in 1 fold and using 0 (k-1) fold for training). Then for the incomplete wing classifier they mention they labelled 1127 images (line 165) but evaluates it on 205 images (what is the train-validation-test split here ?). For the wing classifier, they use a full dataset of 2420 complete wings but a split of 60:20:20 (line 217). Finally, they apply their classifier to "a total of 28,708 wings" (line 310). Overall, I'm a bit lost and I have the feeling we don't have results of the full workflow on a well identified dataset but evaluations of the different steps on distinct subsets. A figure might help to understand the different subsets used as it is currently a bit difficult to draw conclusions for the whole workflow on a well identified dataset. Please clarify.

We thank the reviewer for the comments. We have addressed them below in order of appearance.

- We agree that there are better ways to describe our strategy than one-fold cross-validation. We followed the reviewer's advice and adopted the phrase "single independent train-val-test split".
- We acknowledge that our language here was misleading. We used a sample size of 205 samples to create sample statistics that were used to calculate confidence intervals. The data determined the actual size of the test set split 60-20-20.

We have amended lines 224 to explain our process more clearly; "randomly sampling 205 samples, 5000 times to create sample statistic distributions."

- Regarding the landmark detection model split, we also used a split of 60-20-20. We explain in the implementation details that we use the sample split for all model training. However, the comment refers to a wing classifier. There may have been a mistake; we could not relate this line to the reviewer's comment.
- Regarding the comment on only evaluating our model on distinct subsets, we agree that there is a fair amount of complexity in the workflow of this system. We have replaced figure 1 with a new diagram that more accurately explains the data acquisition and labelling process as well as model evaluations. We hope this helps clear up the process. We want to note that the labelling process is very time-consuming. We collected data independently for each task using the methods we described. To evaluate the overall system on the entire dataset, we empirically evaluate the wing length between the manual recordings done in the lab for each fly. It may be beneficial to take a large subset and label every wing to test the system quantitatively on a distinct labelled set. Along with creating training data, this would take a tremendous amount of effort; we suggest that the results on the test sets created for each task are enough to gauge its performance in deployment.

- Some choices (such as parameter values) are not well motivated: do they come from unbiased empirical evaluation or were these optimized on the test set ?. This should be clarified: Line 216 "we determined the average location of all pixels greater than or equal to the seventh-highest pixel value in the map"; Line 237 "we randomly sampled from -5,5% ... --2°,22°".

We appreciate the reviewer's comment; we realise this is a shortfall. We did not spend time optimising these hyperparameters. Instead, we chose them based on a visual inspection of what degree of augmentation seemed reasonable. Since landmarks are often close to the borders, there was a limitation to how much we could augment the landmarks without them falling outside the image dimensions. We also ran a few trial and error runs to determine suitable ranges after visually inspecting.
Concerning the number we chose for determining the average location, we necessarily care about the highest pixel values; we concidered choosing the seven highest-valued pixels in the segment was a sensible number to estimate the location. We have added to our discussion (on line 507-510) that this is something to experiment with.

- “The segmentation model required a segmentation disk of radius R and used the n_{th} highest pixel values to infer a final landmark location. Both these parameters were not experimented with and could be optimised to improve performance further.”

- In Discussion (line 355), authors compare their approach to other published approaches. It is a bit weak to claim that "our models perform better" because datasets are completely different. One stronger option would be to evaluate these methods on author's dataset, or to evaluate author's methodology to datasets used in these published works. Another option is to avoid such comparison or write it more carefully.

We thank the reviewer for the comment. Ideally, we would apply a transfer learning approach to show the results of this comparison. Unfortunately, adding a transfer learning approach

would require a great deal of time and effort. We chose to restructure our argument and added this comparison to the paragraph about how to use our model on drosophila datasets. We also loosen the argument about performing better than Vandaele et al. and Porto et al. and instead argue that our method could potentially yield better results if transfer learning is successful (line 520-529).

- "Loosely comparing our results with former mentioned studies, Vandaele et al. reported a similar mean pixel distance error of approximately 6 pixels for images of a smaller size 1440X900. Porto et al. reported a mean pixel distance error of 0.57%, normalising the mean pixel distance by the largest wing length. We obtained a smaller normalised pixel distance error of 0.47% for the deployed regression model using the same metric. The Drosophila data sets are arguably less complex than our data, which often contains image artefacts and varying wing quality that may affect the complexity of the task. Therefore we argue that successfully applying transfer learning could yield better results on these Drosophila data sets."

- More source code documentation would be helpful to help others reuse it.

We appreciate the reviewer's suggestion. We have published the code and will add a detailed readme for reproducibility before publishing.

- For a dataset contribution, authors should make it available for reviewers with more detailed description (reference 40 states "TODO" for Data Dryad) and clarify what is the "final dataset" (line 421) and line 444 "resulting landmark data": Do we get images ? How many images ? Is it 28708 ? What are the biological/technical metadata associated to them ? S1 only presents "some of the variables", what are others ? Are all these metadata directly computer-readable ? Do you provide images of the pages or individual wings ... ? Could this dataset also easily be used by other computer scientists developing other landmark detection workflows ?

We thank the reviewer for this comment. We have published our data to the data Dryad which also describes the data in detail. We agree that we still need to explain what is included in the final data set fully; we clarified this on lines 533-537.

- "This data includes the biological recordings for each fly fully described in S2, as well as the associated landmark predictions provided by this study along with all training data and annotations used to train our landmark detection models. We also provide the tsetse fly wing images from volumes 20 and 21 consisting of 14,354 images."

Reviewer 3:

In this work, the authors propose an image processing workflow for the detection of landmarks in Tsetse single-win (RGB) pictures. The number of landmarks (i.e., 11) and their positions were semantically defined. Therefore, they propose using artificial neural networks in two different ways: regression of the coordinates for each of the 11 landmarks or semantic segmentation of the landmarks. They provide an extended assessment of these approaches

by using (1) the classical accuracy metrics and (2) by comparing the distances between landmarks with the distances annotated for each wing by the person who collected the data.

Most important contributions and strengths

- Development of a semi-automated data cleaning method to remove damaged data entries (images containing incomplete fly wings) and correct human registration errors (misaligned catalog pages). The authors estimated the expected results manually and determined that the methods performed well.

- Developed two deep-learning methods to classify tsetse fly wings based on landmarks previously established in the literature. The methods perform well and can be used to distinguish between fly populations coming from different geographic regions.

- The result assessment has been done using a final application perspective: classical accuracy metrics and morphological parameters are evaluated to assess the practicability of the method.

- Python code was made publicly available, is annotated in a comprehensive manner, and is reusable in Jupyter Notebooks. The authors used a publicly available annotated dataset.

Weak points

- Semi-automated data cleaning step to remove images containing incomplete fly wings is based on the detection of 2 out of 11 landmarks. The authors estimate these to comprise ~77% of the total number of incomplete wings, leaving behind an estimated ~23%. This can be improved. Likewise, S3 section shows that the classification models have mostly a perfect performance. Does this mean that these models are only capable of detecting 77% of the damaged wings?

We thank the reviewer for the comment. The reviewer rightly mentioned a few limitations of our study. This indeed means the model can detect an estimated 77% of damaged wings; however, as mentioned on line 314, based on a visual inspection, the classifier removed other categories of incomplete wings as long as landmarks 4 and 6 were missing. Our approach uses the data characteristics to largely solve the problem of removing incomplete wings. A more general approach to detecting incomplete wings is worth further investigation. We mention this as part of our discussion (lines 502-505); however, our solution to the problem adds value to anyone else attempting to build a more robust solution, since the data characteristics explored in the current study shed light on the types of missing wings and their frequency.

- “Future research could improve the current incomplete wing classifier by finding a method to remove all classes of incomplete wings. Alternatively, one could develop a model that predicts landmarks for both complete and incomplete wings, paying particular attention to avoiding wing shape biases in incomplete wings.”

- The catalogue page alignment method is less robust at detecting pages with small misalignments, especially if they do not occur at the beginning of a page.

We agree that despite our best efforts, we might not have caught all misalignments. As the reviewer mentions, the alignment method is less robust in the specific case where pages have a small number of misalignments; hence, we are likely to detect the majority of misalignments. Furthermore, our sample statistics (lines 378-382) suggest that we could detect the expected amount of misaligned pages.

- The authors propose the application of standard deep-learning techniques to build a workflow. Therefore, the current work lacks methodological novelty.

We agree with the reviewer that the deep learning models are not novel. However, we compare them to popular architectures for future work to build from as a benchmark. In addition, our approach is unique to the problem and hence a novel approach concerning the practical problems we had to overcome in this study.

Major comments

Abstract: The information is very complete but I would strongly suggest the authors reorganise it. For example, the information about the number of images could be given together with the size of the images at the end, when results are mentioned.

We agree with the reviewer and moved the information about the size of the data set to the results section of the abstract. We also mention this again in the results section for clarity (line 362).

When the authors say “fully convolutional networks”, they may be referring to “fully connected convolutional neural network”.

We appreciate the reviewer pointing this out. We have checked through the manuscript to ensure we are using the correct term. To our understanding, a convolutional neural network is traditionally a network with convolutional layers (most often including max pooling) that end in fully connected layers or otherwise referred to as a dense layer(s). A fully convolutional network has convolutional layers and no fully connected layers with an activation map output. We sometimes mistakenly use the term “fully convolutional neural network” instead of “fully convolutional network”, which is often how it is referred to in literature. We have renamed all instances of fully convolutional neural network to fully convolutional network or used the acronym FCN.

The current work does not propose a new approach to deal with the unbalance between the background and foreground (weighted loss functions, focal loss, or specific sampling) but it uses the common dice loss function, so I strongly recommend removing the statement about “data-centric approach paying attention to consistent labelling”.

We took the reviewer's advice to remove the statement about the data-centric approach and instead just explained that “we pay particular attention to consistent labelling and data augmentations in training data to improve model performance” in the abstract.

Moreover, data augmentation is a common procedure when training convolutional neural networks, so I would not highlight it in the abstract. The following sentence could be rephrased as the authors do not provide data but a reusable code (“The resulting landmark data set was provided for future morphometric analysis.”).

We thank the reviewer for pointing this out. We have removed the phrase about data augmentation in the abstract. We have also added the landmark data to the Data Dryad, which we referenced in the paper. The resulting landmark data is one of the major contributions of this study.

Moreover, the current work does not provide real units of size (centimetres or millimetres) for the pixels, which could be confusing if used for further biological research.

We thank the reviewer for pointing this out. We have provided the pixel size in millimetres in Fig 1 caption.

Figure 1: The authors say: “The image also contains a scale that can be useful for placing later errors into context” Are the authors referring to the 50 pixels scale bar in the image? If so, this scale bar is virtually added so it does not provide additional value to the framework.

We thank the reviewer for this comment. We have added the pixel size information to the caption. The virtual bar is more for the reader to gauge visually how big the pixel distance errors are, compared to the anatomical features of vein intersections. The relative size of the error may be unclear if we only reported the pixel distance errors; hence we added the scale bar.

Also, the method is proposed to measure wing morphology, so the authors should strongly consider recovering the original pixel size of the images.

We thank the reviewer for pointing this out; we realised we needed to explain our image resizing process clearly. We have more clearly explained this in line 168.

Figure 2: It has poor information to become one single figure. This is a potential proposal to consider: As the authors show the regression approach in this figure, they could merge the information from Fig4 and also show the segmentation approach already shown in Fig3.

We thank the reviewer for this comment. We agree that figure 2 is quite sparse. In fact we realise that the deployment model and models experimented with do not need to feature in this diagram to serve its purpose of explaining the overall workflow. We believe that only the overarching task needs to be depicted, not the details of the methods. In fact we also experimented with more than one classifier but do not include this in the diagram. It has also been noted that it is hard to follow the data acquisition and overall workflow process. So instead we have replaced figure 2 with a more elaborate diagram more clearly explaining the full workflow.

Still, if the authors decide to keep Figs 3 and 4 independently, then Figs 1 and 2 could be merged to provide a summary of the proposal, and Figs 3 and 4 should be more detailed (e.g., number and size of convolutional layers, input and output sizes, whether skip connections exist).

We thank the reviewer for this thoughtful suggestion. Since figure 2 is now more elaborate, we decided to keep figure 1. (the tsetse fly wing image) separately to portray the tsetse fly wing image. We considered adding more detail to figs 3 and 4, but decided not to since these models were standard and thoroughly described in their original publications. We only wish to portray the main differences in model architecture specific to the current study; a pre-trained CNN ResNet with a modified output size and an FCN with an encoder and decoder with a modified input and output size.

The page misalignment problem and its proposed solution is unclear in the text. Adding visual examples or representing this information graphically could be a useful way to make it easier for readers to understand it.

We thank the reviewer for this suggestion. We have taken the advice to create a diagram which can be found in S1. We hope it more clearly explains the problem.

The proposed method performs an image transformation to have the wings always oriented in the same manner. On the other side, the network sees the entire wing in one shot (the input image) and each layer of the output (both for regression and segmentation) belongs to a semantic class (e.g. 5th layer infers landmark number 5). Therefore, the necessity to reorient the wings is not clear. Additionally, should this transformation be done manually one by one as a preprocessing step?

We thank the reviewer for this comment. We have tried to address it below. We hope that it is sufficient. The orientation of features (veins) for each semantic class would only be consistent if we trained it by first converting all images to face the same direction. If we apply the regression network on an image facing the wrong direction, it won't perform very well. We could train the models on both left and right wings, but having left and right wings in the training set slightly increases the complexity. The networks are large enough to perform well on this slight added complexity, but we chose to standardise the process by converting them all to left wings. In addition, the model will perform poorly when an image is facing right, which can happen when we have misalignment, or an image was mislabeled, which in the end, helps us find these types of outliers (e.g. Fig 8 (G)). Furthermore, it is effortless to transform the images because the images are named according to their direction (mentioned in lines 144-145).

The authors adapt the shape of the pictures to the input size of the convolutional neural networks. Usually, they downsample them. How does this affect the disks used for the landmarks in the segmentation approach.

We thank the reviewer for this comment. It is standard to downsample images to some degree of model training as this decreases the complexity of addressing the “curse of dimensionality”. Therefore downsampling helps increase model performance/accuracy. The disk size is chosen based on the relative area around the landmark on the downsampled image. To preserve proportions, we applied the same amount of downsampling to both the size of the disk on the original image and the image itself.

* Evaluation of the Landmark detection:*

While the authors provide a nice assessment of the method using morphological parameters and metrics, knowing whether errors can be negligible or not could be improved by knowing the pixel size.

We thank the reviewer for this comment, we have addressed this and added the pixel size in the caption of the first figure.

Figure 5: It is used to compare both approaches but it is difficult to see if there exists any practical difference in the performance between both (except for the fact that the regression model demands fewer resources). A potential improvement would be to show this data on a logarithmic scale. Additionally, the segmentation model seems to provide more stable results rather than the regression one.

We thank the reviewer for this comment. We agree; there is very little difference between the errors of the compared methods, especially on the scale we use. However, it is still interesting for some to see which landmarks perform worse than others and compare the two models in this way. We argue that the segmentation model has huge outliers (mentioned in Fig 5 caption), which is the instability that we refer to; small perturbations in the input space can cause major errors in some landmark predictions for the segmentation model (lines 437-439). We considered using a logarithmic scale but did not think that this would facilitate the clarity of our argument; we consider the difference between both models negligible, and plotting on a log scale might exaggerate the difference.

- “This implies that small perturbations in the input space can cause major errors in some landmark predictions which we refer to as model instability.”

Method comparison: the authors decide to evaluate the accuracy of the models for each landmark. However, because the 11 landmarks depend on the entire wing (they are not independent), it makes more sense to evaluate how well the method performs for each independent pair of wings of each Tsetse individual, or if not possible, for each wing.

We thank the reviewer for this consideration. In most studies the landmarks are assessed individually. It is true that the model may correlate the landmarks, which seems to be the case for the regression model, but the segmentation model seems to treat the landmarks separately, hence we see extreme outliers for only some landmarks. Since we do not want the model to be biassed toward the shape of the wing, we actually prefer the model to consider landmarks individually. In a sense we are assessing how it performs on different wings (or wing shape) by doing the procrustes analysis. We hope this sufficiently addresses the reviewers' concern.

In Page 11, the authors argue why they think the regression model is more stable than the segmentation. This statement sounds too strong while the evidence from Figure 6 and 5 are not that clear: The segmentation model has way smaller averaged errors than the regression model (approximately 4 pixels). Knowing that a landmark is represented by one single pixel, it is critical to know what is the pixel size to really consider this amount negligible or not.

We agree with the reviewer that whether the error is negligible needs to be clarified. We have included the pixel size in figure 1 caption to help gauge this. We also included a better explanation for why we chose the regression model on lines 371-378.

- "The regression model also shows correlation between the errors of each landmark which helps to identify outliers (damaged wings missed by the classifier) by comparing the wing lengths of the lab recorded wing length (wlm) and calculated wing length from predictions. The segmentation may still produce very accurate predictions for calculating wing length while others can have a very large error as can be noted in Fig 5."

Moreover, the fact that the errors are not correlated with the Procrustes disparity could be interpreted that the method is inferring the landmark positions regardless of the true shape of the wing, so it is agnostic to the wing model used for Procrustes.

We thank the reviewer for this comment. We are not entirely sure we understand the reviewer's proposal, but we have attempted to answer this to the best of our ability below. We use the model to infer only the positions of the landmarks, which are, in turn, used to study the shape of the wing. If the model were to infer the shape instead of the landmarks, we would have to prove that it is inferring the correct shape, which would be very difficult. Suppose there is a relationship between the error of the landmark predictions and the Procrustes disparity. In that case, there is evidence that the model provides landmarks more accurately for specific wing shapes than other wing shapes. We have tried to make this clear on lines 297-302.

- "landmark position and not the wing shape since it is the landmarks that determine the wing shape, and it will be difficult to determine whether the model is inferring the correct wing shape irrespective of the landmark error. If a relationship exist between the error and shape of the wing, then there is evidence that the model is performing better on some wing shapes than others making it impossible to compare different wing shapes in later morphometric analysis."

The authors propose the incomplete wing classifier as a novel application of the task. The models tested have all of them, mostly a perfect performance. The differences are due to the model initialisation or the model instance, which has a random component. In such a case, it is quite interesting to know which model requires larger resources, has fewer parameters, or converges faster.

We appreciate the reviewer's comment and added this information to supplementary material S5 where we compare model performance. We hope this is sufficient.

Minor comments

For the segmentation methods, the authors calculate a disk around the landmark. How is the diameter of this disc calculated? For detection, it is common to use Gaussian disks so the training and loss functions are not that unstable in presence of outliers or small deviations with respect to the natural intersection of veins in the wing.

We thank the reviewer for this consideration. If we used a gaussian disk we would be changing the task from classification to regression, and would most likely use mean square

error which is sensitive to outliers and the same loss function used for the regression network. We were more interested in trying a Fully convolutional network using a segmentation approach which has different inductive biases, than a regression approach which is sensitive toward outliers (at least when using MSE). We thought it would be more beneficial to try two varying approaches with different tasks, fundamentally comparing well known architecture FCN and CNN. The FCN cnn has been traditionally used for segmentation since it provides an output image.

The section “Directions for morphometric studies using these landmarks” is slightly redundant with the content of the main text. It could be more useful to provide a guideline of what is the exact data format that the code can process, what are the considerations to have when taking pictures to the wings so the workflow can be reused, or more tips in terms of exploiting the current method.

We thank the reviewer for this consideration. This comment is somewhat opposing other reviews who have suggested to elaborate and clarify this section more. Our methods are an important contribution but the resulting landmarks of this research are of importance to biologists, so we feel it is necessary to elaborate more on how to use the landmarks at least in the supplementary material.

Consider correcting the colour palette for colorblindness—particularly Fig 7, 8, and S4.1, which give ambiguous information for certain types of colorblindness. Use an online tool such as https://cololoracle.org/ to simulate colorblindness, and refer to https://www.nature.com/articles/d41586-021-02696-z for palette choice. Remember to update legends after changes are made.

We thank the reviewer for this comment. We have used https://cololoracle.org/ to simulate colour blindness on the images. We changed figure 14 in S5 to be colourblind-friendly. After simulating colour blindness for Figures 7 and 8, we found no need to adapt these figures. We hope this is sufficient.

Training images for the wing classification model all come from a subset of the entire dataset corresponding to a specific year (1995). The authors do not address the possibility of the emergence of new variants in later years, to which the trained model would be agnostic.

We appreciate the reviewer’s comment. We aim to predict the location of landmarks while removing any bias toward wing shape (i.e. not correlated to wing shape); we argue that our models will likely still perform well on new variants. We have also elaborated on our explanation for why Vol 20 and 21 contain much of the variation we would expect. We have addressed the emergence of new variants in a later year in lines 497-501 as follows:

“We cannot know how well our model will perform when applied to different species of tsetse, or indeed different populations of G. pallidipes or G. m. morsiatns. However, since we specifically aim at predicting landmarks while removing any bias toward wing shape, and since the data set used contains much of the variance we could expect (refer S3_Figure), we do argue that these models are unlikely to perform noticeably worse.”

*** Small changes and typos***

Please substitute all the Unet by UNet or U-Net.

We thank the reviewer for bringing this to our attention. We have made changes accordingly throughout the paper.

Confidence intervals are sometimes written as CI and others as confidence intervals. Please, be consistent along the manuscript.

We thank the reviewer for bringing this to our attention. We have made the following changes to be consistent throughout the manuscript:

Abstract - " .. (95\% confidence interval [4.4,10.3]) to 5.34 (95\% confidence interval [3,7]) for the regression model. For the segmentation model, data augmentation did not alter the mean pixel distance error of 3.43 (95\% confidence interval [1.9,4.4]) …."

Lines 378 - "We estimated 2% +- 2.56\% of pages .."

Figure 7: in the y axis it is said between landmark 0 and 5 and in the caption, it is said 1 and 6. Please, correct it.

We thank the reviewer for bringing this to our attention. We have corrected Figure 7 accordingly.

Table 1: what are the metrics of wlm? this applies to Figure 7.

We thank the reviewer for bringing this to our attention. We have included the units in Table 1 as well as the Figure 7 x-axis label.

Line 18: "The data form a subset of a larger collection of more than 200,000 pairs of wings" → "The data belongs to a larger collection of more than …"

We thank the reviewer for this comment. We updated the introduction and lost this sentence in the process.

Line 19: "On each intact wing are 11 landmarks, defined…" → On each intact wing 11 natural landmarks are identified as the points of …

We thank the reviewer for this comment and have restructured this information which is now presented as follows on lines 50-53: "We are primarily concerned with solving the technical problem of developing a semi-automated system that assigns Cartesian coordinates to the 11 landmarks for each tsetse wing. Each of these is defined by points of wing vein intersection (Fig. 1), which have been used in previous morphometric studies on tsetse [3-12].

Line 20 – The link for Fig 1 directs the reader to the bottom of the page (possibly linked to the page number) instead of the actual figure.

We double-checked this on two computers and confirmed that it links to the image. We are not sure what the problem here could be. We will note this to the editor to confirm on their side.

Line 30: “Although detecting incomplete wings WAS not of concern in this study, their methods may be good candidates for our task since both tasks aim at wing classification.” To which tasks are the authors referring with “both tasks aim at wing classification”?

We thank the reviewer for bringing this to our attention. We have made changes accordingly on line 66-68.

- “Although detecting incomplete wings was not of concern in this study, their methods may be a good candidate for our task since it also aims at wing classification.”

Line 31 – Replace “Although detecting incomplete wings were not of concern in this study, their methods may be a good candidate for our task since both tasks aim at wing classification.” to “Although detecting incomplete wings was not of concern in this study, the methods used may be a good candidate for our task since it also aims at wing classification.”

We thank the reviewer for bringing this to our attention. We have made changes accordingly on lines 66-68.

Line 38 – The term “Convolutional neural network” is used here for the first time and repeated throughout the manuscript. Consider defining an abbreviation (e.g., CNN) here and use that abbreviation in subsequent mentions.

We appreciate the reviewer's advice and used an abbreviation for Convolutional Neural Network (CNN) and Fully Convolutional Network (FCN).

Line 39: “the best bottleneck features”: for which task?

We thank the reviewer for this comment. We suggest here that convolutional neural networks can learn features from large datasets for both classification and regression tasks. The property is especially leveraged in transfer learning, where the outputs from convolutional layers in a pre-trained CNN are used as bottleneck features for a traditional machine learning model when there is limited data.

We have changed line 74 to make this clear:

“Convolutional neural network architectures are often used on large data sets to automatically learn the best bottleneck features for both clarification and regression tasks …’

We hope this is sufficient.

Line 44: “fully CONNECTED convolutional neural networks.”

We appreciate the reviewer’s comment and have addressed it in an earlier comment as follows: “We appreciate the reviewer pointing this out. We have checked through the manuscript to ensure we are using the correct term. To our understanding, a convolutional neural network is traditionally a network with convolutional layers (most often including max pooling) that end in fully connected layers or are otherwise referred to as a dense layer. A fully convolutional network has convolutional layers and no fully connected layers with an activation map output. We sometimes mistakenly use the term “fully convolutional neural network” instead of “fully convolutional network” which is how it is often referred to in literature. We have renamed all instances of fully convolutional neural network to convolutional network or used the acronym FCN.”

We hope this is sufficient.

Line 46 – The term “Fully convolutional” is used in its hyphenated form in line 44. Check the consistency by using the same form throughout the manuscript.

We thank the reviewer for bringing this to our attention. We have instead chosen to use the acronym FCN (line 81).

Line 51 – Problem in citations. “[?, 20, 21]”.

We thank the reviewer for bringing this to our attention. We have corrected the references mentioned.

Line 82 – The link to “S1 Table” directs the reader to page 7 instead of the actual table.

We thank the reviewer for pointing this out. We will confirm with the editor how this should work since the supplementary material is generally kept in a separate file when published.

Line 83 – Link to Table 1 directs the reader to the bottom of the page (possibly linked to the page number) instead of the actual table.

We thank the reviewer for pointing this out; we have tested it on two computers and have confirmed that the link goes to Table 1. We are not sure why it is not working. We will notify the editor to confirm.

Line 87 – Replace “The distance between landmarks 1 and 6 (refer to Fig 1) was used as this standard measure, converted to a length (wlm) in mm by allowing for magnification differences between microscopes.” by “The distance between landmarks 1 and 6 (refer to Fig 1) was used as this standard measure, converted to a length (wlm) in millimetres, allowing for magnification differences between microscopes.”

We thank the reviewer for bringing this to our attention. We have corrected the sentence accordingly (lines 122-124).

Line 96 – “These volumes are also considered sufficiently large to incorporate much of the variation in all volumes and are suitable for an initial attempt at applying an automatic landmark detection system.”Please provide a brief explanation for the basis of this assumption. Volumes 20 and 21 correspond to only ~7% of the total dataset, which may lead to doubts. Is it possible that new variants emerge throughout the years and consequently their weight is not taken into account from here on?

We thank the reviewer for this comment. “Reviewer 3 is quite correct that volumes 20 and 21 constitute only ~7% of all flies in the parent data set. We accept, therefore, that it is not evident that the subset will “incorporate much of the variation in all volumes. Accordingly, we have added a Figure in supplementary material S3 showing the monthly means of wing lengths across all flies processed. The Figure indicates that, at least, the variation in wing length in volumes 20 and 21 covers most of the seasonal, overall variation in wing length observed in the parent set. Nonetheless, the author’s comment has spurred us to make a softer statement, in which we emphasise the desirability of applying the technique we have developed to all available wings. The sentence in question now reads:

“Volumes 20 and 21 comprise 14,931 pairs of wings, only 7.4% of the 201,545 pairs collected between September 1988 and December 1999. Figure * indicates, however, that

the variation in wing length in volumes 20 and 21 covers most of the seasonal and overall variation observed in the parent set. As such, it seems reasonable to think that the subset is sufficiently representative of the whole to support an initial attempt at developing an automatic landmark detection system. Nonetheless, to further explore the variation in the whole data set, we stress the desirability of applying the technique developed here to all wings available from all 27 volumes. However, this is a major undertaking and was beyond the scope of the present study."

Line 114 – The link for the S2 Table directs the reader to the end of page 17 and not the table.

We thank the reviewer for pointing this out. We will confirm with the editor how this should work since the supplementary material is generally kept in a separate file when published. We have adapted it for easy review to take the reviewer to the correct supplementary material page.

Line 119: "The sample statistics showed that 13% +- 4.63% of wings are incomplete" How was this metric calculated? In particular the standard deviation.

We thank the reviewer for this comment. We sampled from a subset of the data and labelled it with wings to get the proportion of incomplete wings; we then used an online calculator to get the margin of error for the given sample size. Our sample sizes were chosen based on the feasibility of labelling. We use the following confidence interval calculator with the parameters (shown in Supplementary material S5), which also illustrates the formulas used (https://www.calculator.net/sample-size-calculator.html).

Line 121 – Replace "The majority of incomplete wings were missing landmark 4 or 6, making up 10% ± 4.13% of the data, and comprises on average 77% of all incomplete wings." by "The majority of incomplete wings were missing landmark 4 or 6, making up 10% ± 4.13% of the data, comprising on average 77% of all incomplete wings."

We thank the reviewer for bringing this to our attention. We have incorporated this suggestion and moved most of this information to the results section on lines 320-322:
The sample statistics showed that 13%±4.63% of wings are incomplete. Most incomplete wings were missing landmark 4 or 6, making up 10%± 4.13% of the data, comprising 77% of all incomplete wings on average.

Line 122 – Insert quotation marks or use italics in the terms "texitlmkr" and "textitlmkl".

We thank the reviewer for bringing this to our attention. We have made the correction on line 163.

Line 131 – "We then removed incomplete wings using a classification approach. Since most incomplete wings are missing landmark 4 or 6, we trained a classifier to identify wings missing these landmarks.". Training the classifier to detect other landmarks should improve the performance of this task. Assuming the algorithm correctly classifies 100% of the wings lacking landmarks 4 and 6, a remainder of ~23% of incomplete wings are not classified accordingly. Nonetheless, this question is adequately addressed by the authors throughout the manuscript.

We thank the reviewer for this comment. We agree that a classifier or another method can be explored to detect other landmarks. This is a limitation of our study, but our methods largely solve the problem of removing incomplete wings. We hope these methods serve as a foundation for developing better methods.

Line 154 – Correct section title “incomplete wing classifier” to “Incomplete wing classifier”.

We thank the reviewer for bringing this to our attention, and we corrected the mistake.

Line 158 - Replace “Since manually finding training samples is time-consuming, we aided the process by using the biological data set (variables lmkr and lmkl) and then use visual inspection to filter a set of training images.” by “Since manually finding training samples is time-consuming, we aided the process by using the biological data set (variables lmkr and lmkl) and then used visual inspection to filter a set of training images.”

We thank the reviewer for bringing this to our attention. We have made the necessary changes on lines 299-200.

Line 162 – Replace “We removed bad types of examples that do not clearly fall into either class” by “We removed examples that do not clearly fall into either class”.

We thank the reviewer for bringing this to our attention. We have made the necessary changes on lines 203-204.

Line 167 - “We compared three modern computer vision models with transfer learning. These models are ResNet18 [26], Inception [27], and VGG16 [28] with batch normalisation.”. Please provide a brief explanation for the basis of choosing these models specifically.

We thank the reviewer for the comment. We chose these models because they have been used similarly in a previous study on insect wings, mentioned in the introduction on lines 66-68: “With similar wing images, deep learning models have been applied successfully in classifying fruit fly species [11]. Although detecting incomplete wings was not of concern in this study, their methods may be a good candidate for our task since both tasks aim at wing classification.”

We have reiterated this in the “Materials and methods” section on lines 208-211:

- “We compared three modern computer vision models with transfer learning that were used similarly for classifying insect wings in a previous study [11].”

Line 279: “a mean average error (MAE) OF the Euclidean pixel distance”

We thank the reviewer for bringing this to our attention, we have adapted this sentence to the above.

Table 2 – A plot instead of a table could make these results easier to visualise.

Since there are very few data points in this table we prefer to keep it as a table for simplicity and space efficiency. We hope this is sufficient.

Line 361 – “Drosophila data sets are arguably less complex than our data, which often contains image artefacts and varying wing quality which may affect the complexity of the task.” by “Drosophila data sets are arguably less complex than our data, which often contains image artefacts and varying wing quality that may affect the complexity of the task.”

We thank the reviewer for bringing this to our attention, we have adapted this sentence to the above on lines 526-528.

S5: “Alterations to volumes 20 and 21” – Missing spaces between numbers in the “12 pages corrected” line.

We thank the reviewer for bringing this to our attention. We have made corrections.
