# Supplementary material for: Deep learning approaches to landmark detection in tsetse wing images
Source: PLoS Comput Biol. 2023 Jun 26;19(6):e1011194. doi: 10.1371/journal.pcbi.1011194 (PMC10328335; doi:10.1371/journal.pcbi.1011194)
Supplement: Attachment — Submitted filename: Tsetse Addressing reviewer comments 2023.pdf [file pcbi.1011194.s010.pdf]

Addressing reviewer comments 2023

Although Reviewers suggested a minor revision, two of the issues raised are absolutely critical and must be addressed before we consider this submission for publication: the first one relates to reproducibility, as highlighted by Reviewers 1 and 3, and the second one to baseline comparison, as raised by Reviewer 2. Addressing these points may not necessarily require major changes to the manuscript, but must nevertheless be done.

We are grateful to the Editor for granting us a chance to further improve our manuscript and code. We completely agree with both concerns, therefore we improved the code to make it re-usable by other scientists, and performed the baseline comparison presenting it in the main text.

Reviewer 1

While the code has now a DOI and is referenced appropriately, there is still no readme in the GitHub repository and no code changes in 16 months. Thus, usability and reproducibility of this work are severely impacted (as raised by multiple reviewers). Making code available is great, but it only makes sense when others can use it (i.e. executable, documentation, etc).

Thank you, we have revised the codebase, and provide documentation to aid reproducibility.

The authors now briefly touch on the point of “signal-to-noise ratio and data quality”, but again address this very superficially with a computational mindset. My initial point was more with respect to the question of “How good do your data need to be?”

Dear reviewer,

Thank you for your comment, and we apologize for misunderstanding your initial comment. After doing some research, we are still uncertain about your question. Could we ask you to further elaborate on how we might find the signal to noise ratio on our data set, or how we might define how good our data is? We try to provide a response to the best of our knowledge below.

We provide all the specifications we used to capture the image and the parameters (resolution, zoom) with which to capture these images for reproducibility. The images in general are of a high resolution and can be considered to have a high signal to noise ratio. Visual inspection and subjective assessment indicate that other measures of image quality such as contrast, sharpness are generally very good, while we very rarely observe distortion or artifacts. In this paper we did not experiment with adding white noise to understand how the model performs with increased noise which may be studied in future research.

Line 87 - “.” is missing

Thank you, corrected.

Figure 2 – at the end of the paragraph “.” Is in the next line

Thank you, corrected.

Data Description section: “Nonetheless, to further explore the variation in the whole data set, we stress the desirability of applying the technique developed here to all wings available from all 27 volumes of data. This is, however, a major undertaking and was beyond the scope of the present study.” – This paragraph needs to go into discussion.

Thank you for the suggestion. The paragraph has been incorporated into the Discussion (lines 518-520)

line 335 – typo “squared” should be “squared”

Thank you, corrected.

Reviewer 2

I do not understand why authors did not perform the suggested, very basic, evaluation of baseline (mean position prediction for landmark detection, on the same data and protocol as line 230) without giving a convincing argument about not implementing it. Indeed it should take less than one day on the tetse_data.csv: for each landmark compute the mean coordinates values on the learning set, use these mean values as predictor, compute metrics on the test-set, report them similarly as you did for deep learning models. I still believe such a baseline is important when releasing a new dataset. This basic evaluation could strengthen the interest of using their deep learning approaches and let readers better assess how difficult is the task. Instead, authors only replied "The suggestion for trying a model that predicts the mean of landmark positions from the training set is a neat way of comparing how much better the models perform compared to the most straightforward solution. We suggest that the models we present are baseline models, i.e. a good starting point for further development".

We are thankful for the suggestion. We performed the baseline evaluation and added the results to the main text, see lines 228-229, 334-337, Figure 5 and corresponding caption.

Their answer concerning some parameter value choices (Section training implementation, data augmentation) is OK, but this could be translated to an additional sentence in the main text stating that for these parameters "we chose them based on a visual inspection of what degree of augmentation seemed reasonable" (line +/- 280).

Thank you for your suggestion. We added the following sentence to the manuscript at lines 280-281: “We chose these intervals based on a visual inspection of what degree of augmentation seemed reasonable.”

I don't think the abstract and author's summary should mention "The methods we have developed should apply to studying the wings of any insect species" (abstract) neither "Our method applies to the study of the wings of any insect species" (author's summary) as it has not been tested (as recognized later in main text line 570-571). Moreover, although the source code is provided, it is in a state that will not facilitate direct reuse on other data as it is provided as Jupyter Notebook without library versioning (a pip requirements file would be welcome), with specific filenames explicitly mentioned in the source code, and with very little documentation. So I think applying the methodology on other data would not be straightforward.

We agree with the Reviewer and made accompanying changes in our wording throughout the manuscript.

"sqaured" line 335 -> "squared"

Thank you for picking this up, we addressed it.

Reviewer 3

"We agree that despite our best efforts, we might not have caught all misalignments. As the reviewer mentions, the alignment method is less robust in the specific case where pages have a small number of misalignments; hence, we are likely to detect the majority of misalignments. Furthermore, our sample statistics (lines 378-382) suggest that we could detect the expected amount of misaligned pages."

I agree with the authors that having a method that performs accurately for the current task is a good solution. However, I would suggest including further information about the algorithm's limitations in the discussion so other researchers can take it into account

We incorporated and clarified the limitations of the algorithm in the Discussion (lines 478-487) as follows: “Secondly, we cannot ensure that our R^2 threshold method achieves perfect alignment between the images and the biological data, therefore some misalignments might still be present. This is because the threshold method is less robust in the specific case where pages have a small number of misalignments, i.e. pages with only a few misalignments are less likely to be detected since they will most likely still have a high R^2 value. We are more likely to pick up misalignments due to photographing mistakes that happened at the beginning of a page, since this will mean the subsequent flies will not be in the correct order, resulting in a low R^2. However, the sample statistics for misaligned pages indicate that the expected amount was detected, thus the majority of misalignments were found and corrected.”

Figure 2: It is a supportive graphical information to understand the pipeline. If possible, I would suggest the authors switching the order of the tasks (segmentation and classification) to align with the order of the tiers.

Thank you for the suggestion. It has been done in the revised manuscript

Wing reorientation: While it is true that this task is not a milestone of the proposed pipeline, the following statement could be slightly strong. This is particularly important due to the lack of numerical comparison between the non- and reorientation approaches: "The orientation of features (veins) for each semantic class would only be consistent if we trained it by first converting all images to face the same direction. If we apply the regression network on an image facing the wrong direction, it won't perform very well." CNN can learn to be invariant to translation/rotation quite easily and it is indeed a typical feature when analyzing bio-images. Namely, in this case the network sees the entire wing so it should be even easier for the CNN to identify all the landmarks. For this reason, without strong evidences, I would recommend the authors to smooth such statements.

We believe this issue was only discussed in detail in our previous Response letter, rather than in the manuscript. We nonetheless agree with the Reviewer, and we carefully checked throughout the manuscript and made sure we did not use such strong statements without numerical comparisons.

The authors use an online calculator for the sample size estimator (https://www.calculator.net/sample-size-calculator.html). It would be recommendable including this information in the manuscript to ensure reproducibility

Thank you for bringing this to our attention, we have added this information on line 322

“We use an online sample size calculator[66] for these sample statistic calculations.”
